# Graphene Oxide-Graft-Poly(l-lactide)/Poly(l-lactide) Nanocomposites: Mechanical and Thermal Properties

**DOI:** 10.3390/polym9090429

**Published:** 2017-09-07

**Authors:** Li-Na Wang, Pei-Yao Guo Wang, Jun-Chao Wei

**Affiliations:** 1 College of Chemistry, Nanchang University, Nanchang 330031, China; linawang@nit.edu.cn (L.-N.W.); 18942337364@163.com (P.-Y.G.W.); 2 College of Science, Nanchang Institute of Technology, Nanchang 330029, China

**Keywords:** graphene, poly(l-lactide), surface modification, nanocomposite

## Abstract

The surface modification of graphene sheets with polymer chains may greatly hinder its aggregation and improve its phase compatibility with a polymer matrix. In this work, poly(l-lactic acid)-grafted graphene oxide (GO-g-PLLA) was prepared via a simple condensation polymerization method, realizing its dispersion well in organic solvents, which demonstrated that the surface of GO changed from hydrophilic to hydrophobic. GO-g-PLLA can disperse homogeneously in the PLLA matrix, and the tensile test showed that the mechanical properties of GO-g-PLLA/PLLA were much better than that of GO/PLLA; compared with GO, only 3% GO-g-PLLA content can realize a 37.8% increase in the tensile strength for their PLLA composites. Furthermore, the differential scanning calorimetry (DSC) and polarized optical microscopy (POM) results demonstrated that GO-g-PLLA shows a nucleating agent effect and can promote the crystallization of PLLA.

## 1. Introduction

Poly(l-lactic acid) (PLLA), as an important biocompatible and biodegradable polymer, has been widely used in package materials [1], tissue regeneration [2], drug delivery, and many biomedical fields [3]. However, its poor mechanical properties and slow crystallization rate have greatly restricted its applications [4]. In order to overcome this issue, many inorganic fillers, such as bioactive glass nanoparticles [5], hydroxyapatite [6], carbon nanotubes [7], and graphene oxide [8], have been used to reinforce the PLLA matrix and enhance its crystallization rate. However, a vital problem for nanofiller-reinforced composites is that the nanoparticles always aggregate easily in the polymer matrix, and thus the properties of the composites are far from their theoretical values [9,10]. So many works have focused on the surface modification of nanofillers to enhance their phase compatibility and homogeneous dispersion state [11]. For example, grafting polymer chains on the surface of hydroxyapatite [12], as well as the treatment of carbon nanotubes and many other nanofillers have been widely reported [13,14,15].

Graphene, as a kind of novel nanofiller, can significantly improves the mechanical and thermal properties of a polymer matrix [16,17]. However, the strong π-π interaction leads to the intrinsic aggregation of graphene sheets and makes it difficult to disperse homogeneously in a polymer matrix, and thus many methods including covalent and noncovalent surface modification of graphene sheets have been designed to tune its surface properties and phase compatibility with polymers [16,17,18]. Up to now, several reports on PLLA-grafted graphene hybrids have been reported. He et al. used the in situ ring opening polymerization of d-lactide on the surface of GO sheets and prepared poly(d-lactide)-grafted graphene (GO-g-PDLA) [19]. GO-g-PDLA can form stereo-complex with PLLA and thus enhance the phase interaction between GO sheets and PLLA chains, in addition to exhibiting an effective nucleating agent effect. Lee’s group also used the in situ ring opening polymerization method to prepare graphene oxide/PLLA composites with PLLA chains grafted on the surface of GO sheets [20]. However, the in situ grafting process happens in a rigid anhydrous and inert environment, and is furthermore a time-consuming process. Xu’s group prepared PLLA-grafted graphene oxide via a melting polycondensation method of GO and l-lactic acid monomer; in this method, a catalyst was used, and the reaction was carried out at a high temperature (180 °C) [8].

Generally, to prepare PLLA chains, three methods are always used; in situ ring opening polymerization, melting polymerization, and polycondensation in the solvent. Each method has its advantages and drawbacks. Polycondensation of l-lactic acid in the solvent is a convenient method to prepare PLLA chains, as this method is simple, does not require a rigid anhydrous condition, and is a catalyst-free and relatively low-temperature process, but this method cannot form high molecular weight PLLA chains. However, it is a very convenient method to graft oligo PLLA chains on the surface of nanoparticles. For example, Chen’s group used this method to prepare PLLA-grafted hyroxyapatite nanoparticles, and the results demonstrated that the oligo PLLA is enough to tune the surface properties of nanofillers [21,22].

In this manuscript, PLLA-grafted graphene oxide (GO-g-PLLA) was prepared via the simple condensation polymerization method, and the reaction was conducted at 120 °C, much lower than the reported 180 °C [8]. Furthermore, no catalyst was used in this method. The mechanical and thermal properties of GO-g-PLLA/PLLA composites were investigated, and the results showed that the mechanical properties of Go-g-PLLA/PLLA composites were much better than those of GO/PLLA composites. Moreover, the GO-g-PLLA hybrids can work as nucleating agents and promote the crystallization of PLLA.

## 2. Experimental

### 2.1. Materials

GO was prepared by the oxidation of graphite according to the modified Hummers method [23]. PLLA (revode 190) and l-lactic acid was donated from Hisun Biomaterials Co., Ltd. (Taizhou, Zhejiang, China).

### 2.2. Preparation of GO-g-PLLA

Firstly, two hundred milligrams of GO were dispersed homogeneously in 10 mL of H_2_O, and then 600 mg l-lactic acid and 350 mL toluene were mixed with the GO solution. The mixture was heated at 120 °C for 12 h under vigorous stirring; in the meantime, the water was removed with the boiling of toluene. In the end, the solid product was collected by centrifugation, and washed with chloroform several times. The final product (GO-g-PLLA) was dried under vacuum at 40 °C.

### 2.3. Preparation of GO-g-PLLA/PLLA Nanocomposites

The GO-g-PLLA/PLLA nanocomposites were prepared via a solvent mixing method. Briefly, PLLA and GO-g-PLLA were dissolved in chloroform, respectively, and then PLLA and the GO-g-PLLA solution were mixed together under vigorous stirring. The mixed solutions were poured into a glass dish, when the solvent evaporated completely, the GO-g-PLLA/PLLA nanocomposite film was obtained.

Composites with different GO-g-PLLA content, such as 0.3, 0.7, 1.0, 3.0, 4.0, and 5.0 wt % were prepared, and the corresponding composites are denoted as GO-g-PLLA0.3/PLLA, GO-g-PLLA0.7/PLLA, GO-g-PLLA1/PLLA, GO-g-PLLA3/PLLA, and GO-g-PLLA5/PLLA, respectively.

### 2.4. Characterizations

The Fourier transform infrared (FTIR) spectra of GO and GO-g-PLLA were recorded at room temperature in a Shimadzu IRPrestige-21 Fourier transform infrared spectra-photometer.

The X-ray photo-electron spectroscopy (XPS) of GO and GO-g-PLLA was obtained with a thermo-VG Scientific ESCALAB 250 photo-electron spectrometer using a monochromatic Al Ka (1486.6 eV) X-ray source.

The atomic force microscopy (AFM) of GO and GO-g-PLLA was measured on a nanoscope III A (Digital Instruments) scanning probe microscope via the tapping mode.

Raman spectra of GO and GO-g-PLLA were acquired using a Raman spectrometer (Horiba Jobin Yvon) operating with an Xplora microraman system. 

The thermogravimetric analysis (TGA) measurements were performed by PyrisDiamond TG/DTA. The samples were heated from room temperature to 800 °C at a rate of 10 °C/min under nitrogen flow.

The dispersion test of GO and GO-g-PLLA in chloroform and water were measured at 2 mg/mL concentration. The samples were dispersed in solvent by sonication and then the optical photos were taken with a general digital camera. The tensile tests of GO/PLLA and GO-g-PLLA/PLLA nanocomposites were measured on an electronic universal testing machine (CMT8502, Shiji Tianyuan Instrument Co., Ltd., Shenzhen, China) at a tensile speed of 10 mm/min at room temperature. The test of each sample type was repeated three times. The morphologies of tensile fractured surfaces were observed by scanning electron microscopy (SEM, QuanTA-200F, FEI Company, Hillsboro, OR, USA).

Thermal analysis of neat PLLA and GO-g-PLLA/PLLA was conducted using a TA Instruments differential scanning calorimeter (Q2000, TA instruments company, New Castle, DE, USA) under nitrogen purge. For non-isothermal melt crystallization, the samples were heated from room temperature to 200 °C at a heating rate of 10 °C/min, and kept for 3 min, cooled to 20 °C at 10 °C/min, and then heated to 200 °C at 10 °C/min.

The crystalline morphology of neat PLLA and its nanocomposites were observed via a Nikon E600 polarized microscope (POM) equipped with a temperature controller (Hs400, Instec Company, Boulder, CO, USA). The samples were first heated at 190 °C for 3 min and cooled to 130 °C at a cooling rate of 100 °C/min^−1^, then isothermally crystallized for 50 min.

Wide angle X-ray diffraction (WAXD) patterns of GO, PLLA, and PLLA/GO-g-PLLA nanocomposites were acquired with a D8 focus X-ray Diffractometer operating at 30 kV and 20 mA with a copper target (λ = 1.54 Å). The scanning range was from 5° to 40°, and the scanning speed was 2° min^−1^ at room temperature.

## 3. Results and Discussion

### 3.1. Synthesis and Characterization of GO-g-PLLA

**Figure 1:**
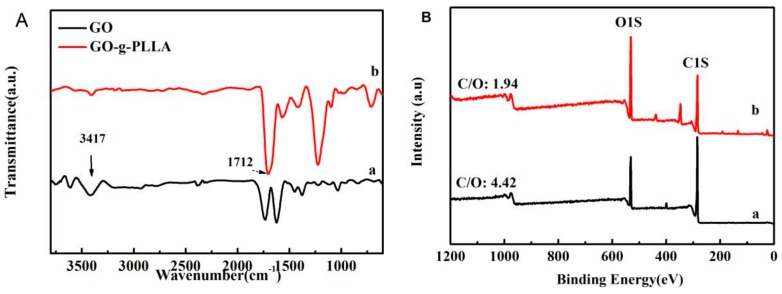
Fourier transform infrared spectra (**A**) and X-ray photo-electron spectroscopy; (**B**) spectra of GO (a) and GO-g-PLLA (b).

As shown in Figure 1A, curve (a), the characteristic peaks at 3417 and 1750 cm^−1^ are attributed to the stretching vibration of the hydroxyl groups and carboxyl groups of GO, which are reacted with the carboxyl or hydroxyl groups of lactic acid, respectively. When GO was mixed with l-lactic acid at a higher temperature, the polycondensation of l-lactic acid occurred; at the same time, the l-lactic acid monomer or oligo-polymers also reacted with the hydroxyl or carboxyl groups of GO, and thus poly(l-lactic acid) chains were grafted on the surface of the GO sheets. Compared with the IR spectra of GO, the intensity of absorption peaks at 3400 and 1750 of GO-g-PLLA (shown in Figure 1A, curve (b)) was much weaker, while a new peak appeared at 1712 cm^−1^. This new peak is attributed to the stretch vibration of ester groups in PLLA chains, demonstrating that PLLA was successfully grafted on the surface of GO.

**Figure 2:**
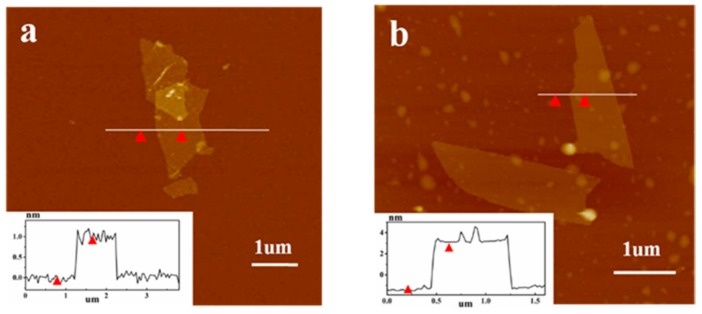
Atomic force microscopy images of GO (**a**) and GO-g-PLLA (**b**).

When PLLA chains were grafted on the surface of GO, the surface composition of GO changes considerably. As shown in Figure 1B, two typical peaks at 288 eV and 532 eV were recorded, which are the characteristic peaks of C1s and O1s. The C/O ratio of GO was 4.42, while the C/O ratio of GO-g-PLLA was 1.94, which is much lower than that of GO, indicating the increase of oxygen content, and this change was due to the higher content of oxygen in PLLA chains. On the other hand, the existence of PLLA chains on the GO sheet may also change its thickness, as shown in Figure 2; the average height of a GO sheet is about 1.0 nm, while the average height of GO-g-PLLA is about 3.8 nm, so it can be judged that the thickness of the PLLA layers may be 2.8 nm.

**Figure 3:**
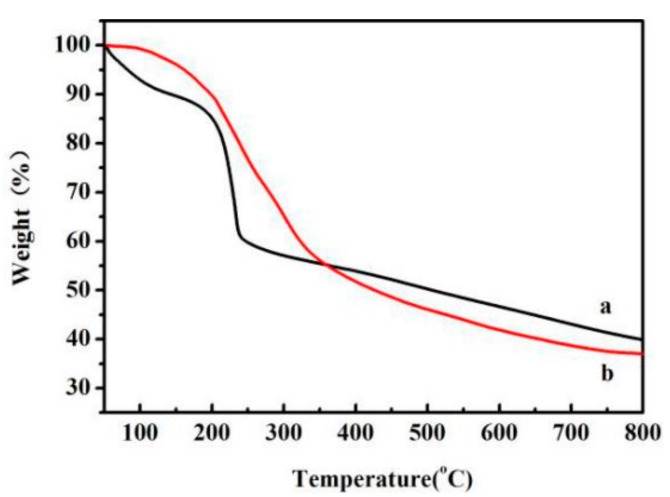
Thermal degradation curves of GO (**a**) and GO-g-PLLA (**b**).

The TGA curves of GO and GO-g-PLLA are shown in Figure 3. The weight loss below 150 °C was ascribed to the evaporation of adsorbed solvent; because of the strong hydrophilic properties of GO, it can easily adsorb water in the air, and even if it is lyophilized a long time, it is still difficult to remove the adsorbed water. On the contrary, GO-g-PLLA is hydrophobic due to the existence of PLLA chains, and thus it is not easy to absorb water. Therefore, in the TGA test, the weight loss of GO-g-PLLA is much lower than that of GO. With the increase of temperature, the surface functional groups such as hydroxyl and carboxyl groups begin to detach from the GO sheet, especially when the temperature is above 200 °C, causing the GO sample to show a quick weight loss. The total weight loss of GO in the range between 150 °C and 800 °C was about 49.6%. As for the GO-g-PLLA samples (Figure 3, curve (b)), the weight loss may derive from both the decomposition of the polymer chains and the functional groups of the GO sheet. In the range between 150 °C and 800 °C, the weight loss of GO-g-PLLA was about 59.4%, much higher than that of the GO sample, confirming that PLLA was successfully grafted on the surface of GO. However, the TGA curve of GO-g-PLLA is different from the reported results of PLLA, which shows a decomposition temperature around 330 °C [8]. Because the PLLA chains grafted on the surface of GO were oligo-chains, the molecular weight is much lower than that of the commercial PLLA samples. Thus, GO-g-PLLA decomposed from 200 °C, a relative lower temperature than PLLA, and this result is similar with the decomposition of oligo PLLA-grafted hydroxyapatite [21].

**Figure 4:**
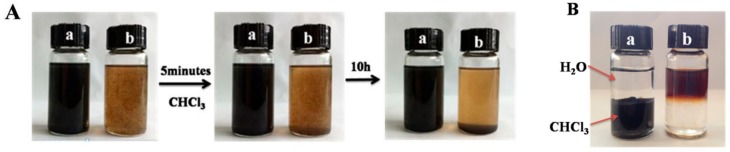
Photos of GO-g-PLLA (a) and GO (b) dispersed in chloroform (**A**) and a water-chloroform two-phase solvent (**B**).

The main purpose of grafting PLLA on the surface of GO is to tune its surface properties, especially wettability. GO is hydrophilic, while PLLA is hydrophobic, so GO-g-PLLA can also exhibit a hydrophobic surface, which can be verified by the sedimentation experiment. As shown in Figure 4, when GO and GO-g-PLLA were dissolved in chloroform, respectively, the GO-g-PLLA could disperse homogeneously (2.0 mg/mL) and the suspension could keep stable for a long time; in fact, 10 h later, the GO-g-PLLA was still dispersed well in the solvent. On the contrary, GO could not enter chloroform, and 5 min later, most of the GO sheets had precipitated at the bottom of the bottle. To further show the change of surface properties, GO and GO-g-PLLA were dispersed in a two-phase solvent composed of chloroform and water (*v*/*v* 1:1). As shown in Figure 4B, GO-g-PLLA dispersed well in chloroform and could not enter water phase, showing its hydrophobic properties. As a control, owing to its hydrophilic properties, GO dispersed homogeneously in the water and could not enter the chloroform phase. These results demonstrated that the surface of GO changed from hydrophilic to hydrophobic by grafting PLLA chains, which may further improve its phase compatibility with organic polymers.

### 3.2. Mechanical and Thermal Properties

**Figure 5:**
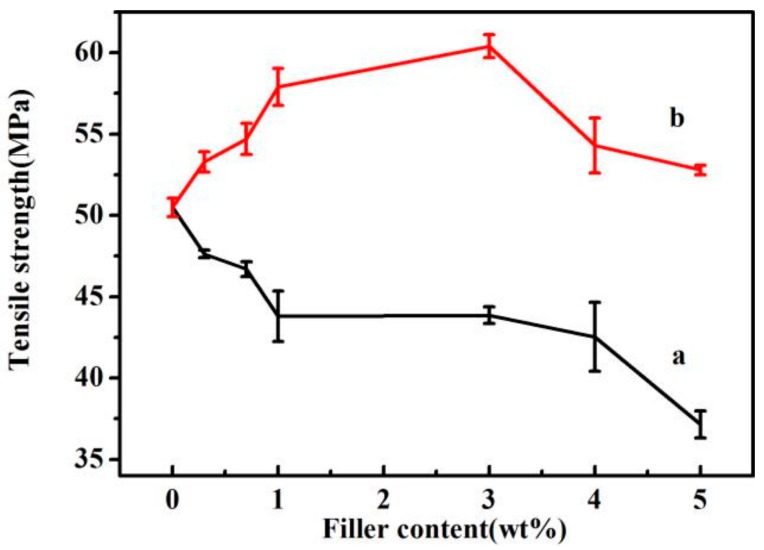
Tensile strength of GO/PLLA (**a**) and GO-g-PLLA/PLLA (**b**) at different filler contents.

When nanofillers are added into a polymer matrix, the mechanical properties of polymers should be reinforced; however, due to the aggregation of nanofillers, the mechanical properties of the composites are always far from their theoretical values, sometimes becoming even worse than the pure polymers. When GO was mixed with PLLA, the aggregation of GO may have reduced the mechanical properties of PLLA. As shown in Figure 5, the tensile strength of PLLA was about 51 MPa; when GO was added, the tensile strength of the GO/PLLA composites was lower than that of pure PLLA. When GO-g-PLLA was used as a nanofiller, the surface of GO was connected with PLLA chains via covalent bonds, and thus the phase compatibility of GO-g-PLLA with PLLA was much better. When exterior forces were added to the composites, the interior stress may transfer easily from PLLA chains to GO sheets, and thus the tensile strength of GO-g-PLLA/PLLA nanocomposites was much higher than that of PLLA and GO/PLLA nanocomposites (shown in Figure 5). For example, when 3.0 wt % of GO-g-PLLA was added into the PLLA matrix, the tensile strength of the composite was 60 MPa, about 37.8% higher than that of GO/PLLA composites.

**Figure 6:**
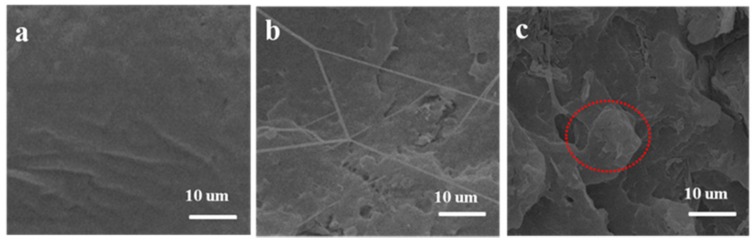
Scanning electron microscopy (SEM) images of the tensile failure section for PLLA (**a**), GO-g-PLLA3/PLLA (**b**) and GO-g-PLLA5/PLLA (**c**). Images in the red circle demonstrates the aggregation of nanofiller.

The tensile fracture surface of PLLA, GO-g-PLLA 3, and GO-g-PLLA 5 are shown in Figure 6. The tensile fracture surface of PLLA (Figure 6a) was much smoother, which is in accordance with the brittle characteristic of PLLA. When GO-g-PLLA was mixed with PLLA, due to the interface interaction, the tensile strength increased, the tensile fracture surface was coarse, and some fibers were observed on the fracture surfaces of GO-g-PLLA3/PLLA nanocomposites (Figure 6b). This may result from the stress transfer from PLLA to GO sheets, confirming that the phase compatibility of GO-g-PLLA with the PLLA matrix was very good and the phase interaction was strong. However, with the increase of the GO-g-PLLA content, the fillers may also aggregate in the polymer matrix, and thus lead to the decrease of the tensile strength (Figure 5). As shown in Figure 6, the tensile surface of Go-g-PLLA5/PLLA was much coarser than that of the PLLA sample, but no fibril structure appeared; only some aggregates were found, implying that GO-g-PLLA did not disperse well. However, our primary test showed that the surface-grafted PLLA chains can change the surface wettability of GO from hydrophilic to hydrophobic, and thus they can enhance the phase compatibility between the PLLA matrix and GO fillers; therefore, it may be deduced that if the composites preparation method was optimized, much better mechanical properties may be obtained. 

**Figure 7:**
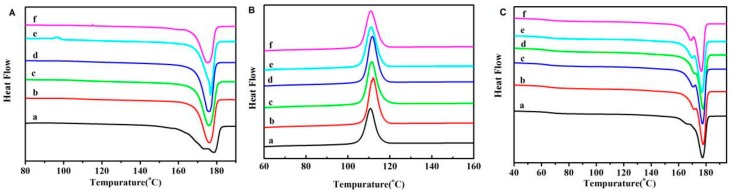
Differential scanning calorimetry (DSC) curves of the first heating process (**A**), cooling process (**B**), and the second heating process (**C**) for different samples. PLLA (a), GO-g-PLLA0.3/PLLA (b), GO-g-PLLA0.7/PLLA (c), GO-g-PLLA1/PLLA (d), GO-g-PLLA3/PLLA (e), and GO-g-PLLA5/PLLA (f).

The thermal parameters of neat PLLA and GO-g-PLLA/PLLA nanocomposites were measured with DSC by a heating-cooling-heating process. The crystallinity (*X_c_*) of different samples were acquired from the DSC curves of the first heating process (Figure 7A) by the following equation:

$$X_{c}=\frac{\Delta H_{m}}{f\times \Delta H^{0}}\times 100\%$$

where $\Delta H_{m}$ is the melting enthalpy of PLLA or GO-g-PLLA/PLLA composites, $\Delta H^{0}$ (93.0 J/g) is the melting enthalpy of PLLA with 100% crystalline [24], and f is the content of GO-g-PLLA in the composites. 

As shown in Table 1, the crystallinity of PLLA was about 52.9%. With the addition of GO-g-PLLA, the GO-g-PLLA worked as a nucleating agent and enhanced the crystallization PLLA, and thus the crystallinity of the PLLA in the composites increased. However, when the filler content was higher than 3 wt %, the crystallinity showed a slight decrease; this may have resulted from the aggregation of nanofillers in the PLLA matrix, as too many aggregates will hinder the arrangement of PLLA chains and thus have a negative effect on its crystallization process. As mentioned in the mechanical properties, the GO-g-PLLA 3 has the highest tensile strength, and this can also be explained by its high crystallinity.

The crystalline temperature (*T_c_*, the peak temperature of the exothermic curves obtained during the cooling process), melting temperature (*T_m_*, the peak temperature of the second heating process), and glass transition temperature (*T_g_*) can be measured from the DSC curves of the cooling process and the second heating process (Figure 7B,C); the corresponding results are shown in Table 1. The *T_c_* and *T_m_* of PLLA was 110.4 and 175.1 °C, respectively, while the *T_c_* and *T_m_* of GO-g-PLLA/PLLA shifted to a higher temperature, indicating the nucleating effect of GO-g-PLLA. The *T_g_* of the GO-g-PLLA/PLLA nanocomposites was lower than that of neat PLLA, demonstrating that the polymer chain fragments in GO-g-PLLA/PLLA composites are much more easily moved than that in the pure PLLA matrix, perhaps because the filling of GO-g-PLLA decreased the entanglements of PPLA chains. Furthermore, the melting peaks in the second heating process were a little different from the first heating process. There were weak shoulder peaks in the curves (Figure 7, curve (c)); in other words, two melting peaks were found. This finding of two melting points for PLLA samples has also been observed in many other references [25,26], and this phenomenon can be ascribed to the differences of crystal morphology. As a matter of fact, when PLLA samples crystallize at a faster speed, there will be more uncompleted crystalline phase, which will melt at a lower temperature. When nanofillers were blended with PLLA, the PLLA composites crystallized more quickly than pure PLLA samples, and thus some uncompleted crystalline formed. Hence, in the second heating process, there were minor melting peaks ascribed to the uncompleted crystals. Moreover, the first melting peaks of the composites are much clearer than that of the PLLA sample. All of these results demonstrated that GO-g-PLLA and PLLA may have strong interactions and a positive effect in enhancing the crystalline speed of PLLA.

**Figure 8:**
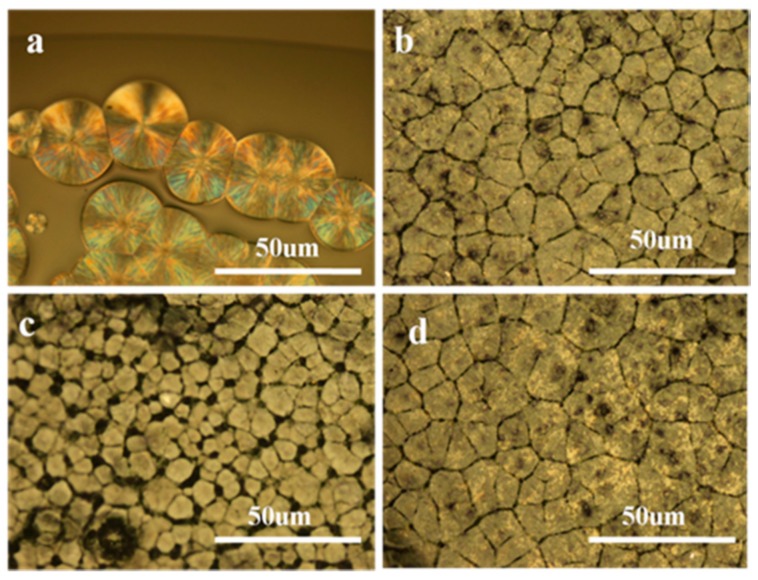
Polarized microscope (POM) images of PLLA and its nanocomposites isothermally crystallized at 130 °C. Neat PLLA (**a**), GO-g-PLLA1/PLLA (**b**), GO-g-PLLA3/PLLA (**c**), and GO-g-PLLA5/PLLA (**d**).

The spherulite morphology of PLLA and its nanocomposites isothermally crystallized at 130 °C were observed with POM. The well-developed PLLA spherulites grew to about 25 μm with clear boundaries (Figure 8a). However, the diameter of GO-g-PLLA/PLLA composites spherulites were smaller than that of PLLA, while the number of spherulites was much greater than that of PLLA, which further confirmed that GO-g-PLLA may act as a nucleating agent that can largely enhances PLLA molecular chain mobility and increases the number of nucleation sites (Figure 8b–d). However, it should be noted that the size of PLLA spherulites first decreased and then increased with increasing the GO-g-PLLA loading, which indicated that the nucleation sites decreased with further increasing the content of GO-g-PLLA (Figure 8d), this may resulted from the aggregation of GO-g-PLLA filler.

**Figure 9:**
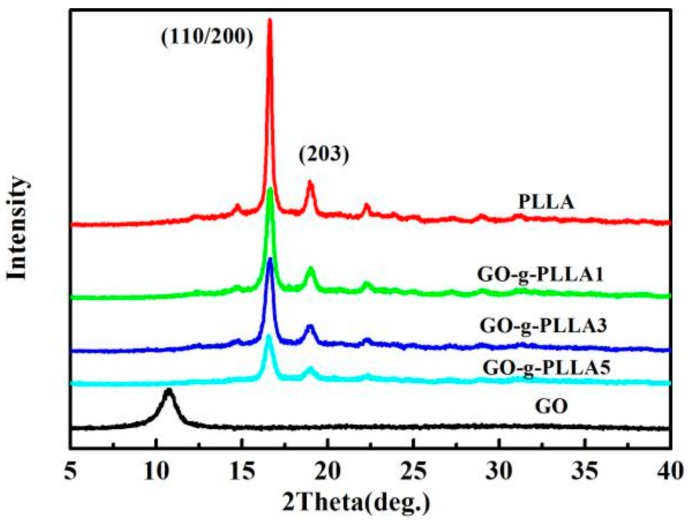
Wide angle X-ray diffraction (WAXD) patterns of neat PLLA and its nanocomposites.

The effect of GO-g-PLLA on the crystal structure of PLLA was detected by WAXD. Figure 9 shows the patterns of neat PLLA, GO, GO-g-PLLA1/PLLA, GO-g-PLLA3/PLLA, and GO-g-PLLA5/PLLA. For neat PLLA, two sharp characteristic peaks are located at 16.6°and 18.9°, corresponding to (200)/(110) and (203) planes, which demonstrates the crystallization in α form. For GO-g-PLLA/PLLA composites, the diffraction patterns are the same as those of neat PLLA. This indicated that incorporation of GO-g-PLLA does not alter the crystalline structures of PLLA in the GO-g-PLLA/PLLA nanocomposites. In addition, the crystal peak of GO is present at about 2θ = 10.8, suggesting that the GO was exfoliated from graphite. On the other hand, in the composite, no GO diffraction peaks were found, and this confirms that the GO-g-PLLA dispersed well in the composites, and did not form ordered arrangements.

## 4. Conclusions

In this work, PLLA-grafted graphene oxide (GO-g-PLLA) was fabricated via a simple condensation polymerization method with GO and l-lactic acid monomer, and used as a nanofiller to reinforce PLLA. The surface-grafted PLLA chains cause the GO surface to transform from hydrophilic to hydrophobic, and render it able to disperse well in chloroform. The SEM, WAXD, and tensile test results showed that GO is exfoliated and uniformly dispersed in the PLLA matrix, which demonstrated that surface modification is an effective method to improve the interfacial interactions between nanofillers and a polymer matrix, showing a much better reinforcing effect than GO alone. The non-isothermal melting crystallization behavior and spherulite morphology observation demonstrated that the GO-g-PLLA may act as a nucleating agent and improve the crystallization speed of PLLA.

## Figures and Tables

**Table 1:** Summary of the thermal parameters of PLLA and GO-g-PLLA/PLLA nanocomposites.

Samples	*T_g_* (°C)	*T_c_* (°C)	*T_m_* (°C)	*X_c_* (%)
PLLA	66.1	110.4	175.1	52.9
GO-g-PLLA0.3/PLLA	63.7	112.1	176.1	56.7
GO-g-PLLA0.7/PLLA	63.1	111.4	176.3	57.0
GO-g-PLLA1.0/PLLA	63.5	111.6	176.2	62.1
GO-g-PLLA3.0/PLLA	62.4	111.2	176.9	61.0
GO-g-PLLA5.0/PLLA	62.7	111.1	175.6	59.1
